# Aging of neural stem cells and vascular dysfunction: mechanisms, interconnection, and therapeutic perspectives

**DOI:** 10.1016/j.cccb.2025.100402

**Published:** 2025-10-10

**Authors:** Khrystyna Duve, Volodymyr Lushchak, Svitlana Shkrobot

**Affiliations:** a I. Horbachevsky’s Ternopil National Medical University, Neurology Department, Troleibusna street, 14, Ternopil, 46027, Ukraine; b Vasyl Stefanyk Precarpathian National University, Department of Biochemistry and Biotechnology, 57 Shevchenko Str., Ivano-Frankivsk, 76018, Ukraine; c Research and Development University, 13А Shota Rustaveli Str., Ivano-Frankivsk, 76018, Ukraine

**Keywords:** Neural stem cells (NSCs), Vascular dysfunction, Neurovascular unit, Oxidative stress, Chronic inflammation, Blood–brain barrier (BBB), Mesenchymal stem cells (MSCs), Cerebrovascular aging

## Abstract

**Background:**

Aging of the central nervous system is accompanied by a progressive decline in neuroplasticity, regenerative capacity, and vascular integrity. Neural stem cells (NSCs) and cerebral vasculature are both critically involved in maintaining homeostasis, and their age-related dysfunction contributes to neurodegenerative and cerebrovascular diseases.

**Objective:**

This article explores the mechanisms underlying NSC aging and vascular deterioration, their mutual interconnection, and the implications for brain health in aging populations.

**Methods:**

We review current evidence on molecular and cellular pathways affecting neurogenesis and cerebral perfusion, including dysregulation of BDNF, VEGF, mTOR, AMPK, and the effects of oxidative stress and chronic inflammation. We also highlight emerging biomarkers and therapeutic targets.

**Results:**

Aging disrupts the neurovascular unit, impairing both neurogenesis and vascular support systems. Chronic inflammation and oxidative stress exacerbate these processes, forming a self-perpetuating degenerative cycle. Antioxidant and anti-inflammatory therapies, along with stem cell–based interventions such as mesenchymal stem cells (MSCs) and induced pluripotent stem cells (iPSCs), show promise for restoring neural and vascular function.

**Conclusion:**

Addressing both NSC and vascular aging is essential for developing effective interventions against cognitive decline and cerebrovascular pathology. Integrative and personalized therapeutic strategies targeting shared molecular pathways hold great promise for future clinical applications.

## Highlights

• Neural stem cell aging impairs neurogenesis via BDNF and VEGF pathway decline.
• Vascular dysfunction promotes BBB breakdown and cerebral hypoperfusion.
• Oxidative stress and inflammation drive neurovascular unit degeneration.
• mTOR hyperactivation and AMPK suppression accelerate NSC senescence.
• Stem cell-based approaches (MSC, iPSC) hold promise for neurovascular repair.

## 1 Introduction

Neural stem cells (NSCs) represent a distinct population of stem cells characterized by their capacity for self-renewal and multilineage differentiation into neuronal and glial cell types, including neurons, astrocytes, and oligodendrocytes. These cells play a fundamental role in sustaining neuroplasticity and facilitating the repair and regeneration of neural tissue, particularly within the adult central nervous system [[1], [2], [3]].

Aging induces profound morphofunctional alterations in the brain, characterized by a progressive decline in cognitive functions, neuroplasticity, and regenerative capacity. A critical contributor to these processes is the dysfunction of neural stem cells (NSCs), which play a pivotal role in sustaining neuronal homeostasis, facilitating synaptogenesis, and maintaining the structural and functional integrity of the central nervous system (CNS) [4,5].

Concurrently, age-related alterations in the cerebrovascular system significantly contribute to the progression of cognitive impairment, neurodegeneration, and cerebrovascular diseases. Reduced angiogenesis, endothelial dysfunction, disruption of the blood-brain barrier (BBB), hypoxia, chronic inflammation, and oxidative stress are interconnected pathological processes that collectively drive the gradual atrophy of brain tissue [[6], [7], [8]].

Recent studies have revealed complex interactions between these processes, where impaired neurogenesis and vascular dysfunction act synergistically through shared molecular pathways, including BDNF, VEGF, mTOR, AMPK, and inflammatory mediators [[9], [10], [11], [12], [13]]. This intricate crosstalk contributes to the disruption of the neurovascular niche and accelerates neurodegeneration.

**Fig. 1:**
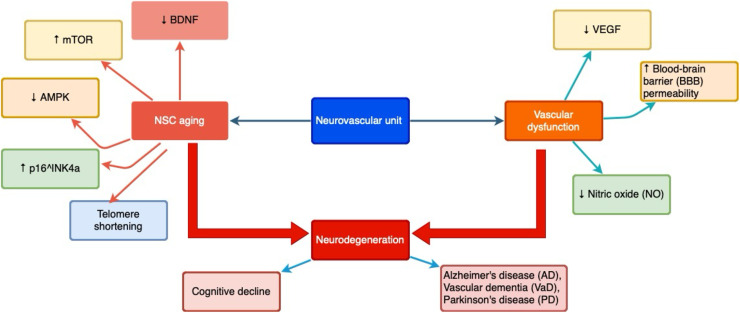
Schematic overview of the interconnection between aging neural stem cells (NSCs) and vascular dysfunction.

It has been established that the synergy between NSC aging and vascular dysfunction plays a pivotal role in the pathogenesis of neurodegenerative and cerebrovascular disorders, including Alzheimer’s disease (AD), Parkinson’s disease (PD), multiple sclerosis (MS), vascular dementia (VaD), chronic cerebral ischemia, and the sequelae of neurotrauma [14]. Investigating the mechanisms underlying NSC and vascular aging provides novel therapeutic prospects for the prevention and treatment of age-related neurodegenerative processes [[15], [16], [17]]. A schematic representation of the interplay between NSC aging and vascular dysfunction and their contribution to neurodegeneration is shown in Fig. 1.

## 2 Aging of neural stem cells and vascular dysfunction

### 2.1 Impairment of neurogenesis

One of the most critical aspects of brain aging is the decline in neural stem cell (NSC) activity within the subventricular zone (SVZ) of the lateral ventricles and the subgranular zone (SGZ) of the hippocampus. This decline is accompanied by a reduced capacity for NSC proliferation and differentiation, leading to a diminished neuronal pool and restricted potential for neuroplastic remodeling of the brain [18]

The key mechanisms underlying this process include:

• Decreased levels of BDNF (brain-derived neurotrophic factor), a key neurotrophin responsible for supporting neuronal survival, promoting synaptic plasticity, and facilitating neurogenesis, result in compromised neuronal maintenance, reduced adaptability of neural circuits, and progressive cognitive decline ([11]; Wang et al., 2022).
• A reduction in VEGF (vascular endothelial growth factor), a key regulator of angiogenesis and vascular homeostasis, leads to impaired blood vessel formation, decreased capillary density, and consequently, chronic cerebral hypoxia, which exacerbates neurodegenerative processes and cognitive decline. VEGF also directly influences the NSC niche, and its deficiency may impair neurogenesis [9,10].
• Hyperactivation of mTOR (mechanistic target of rapamycin), a central regulator of cellular growth and metabolism, leads to the inhibition of autophagy, resulting in the accumulation of damaged proteins and organelles, ultimately accelerating cellular aging and contributing to neurodegenerative processes ([13,19]; Johnson et al., 2018).
• Suppression of AMPK (AMP-activated protein kinase), a key energy sensor that regulates cellular metabolism and stress adaptation, leads to a decline in cellular energy homeostasis, reduces the ability of cells to respond to metabolic stress, and promotes the accumulation of dysfunctional mitochondria, ultimately exacerbating neurodegenerative processes [25,26].
• Oxidative stress induced by NADPH deficiency leads to mitochondrial damage, activation of apoptotic pathways, and impairment of antioxidant enzyme function, thereby increasing neuronal susceptibility to degeneration and exacerbating age-related neurodegenerative disorders [27,28].

Importantly, recent studies highlight that these signaling pathways are not isolated but interact at multiple regulatory levels. For example, oxidative stress and mitochondrial dysfunction activate mTOR and suppress AMPK signaling, while inflammatory cytokines such as TNF-α may downregulate BDNF expression, further impairing synaptic plasticity and neurogenesis. This complex interplay promotes cumulative damage within the NSC niche during aging ([29].

**Fig. 2:**
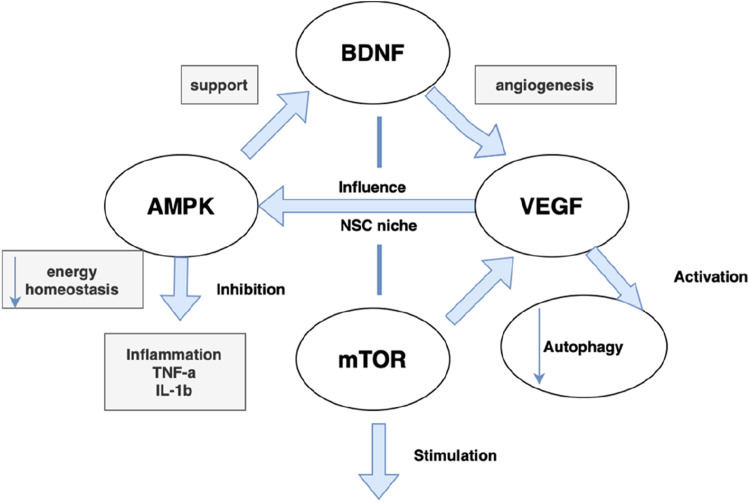
Molecular crosstalk between BDNF, VEGF, AMPK, and mTOR pathways in neural stem cell and vascular aging.

Recent advances have also identified molecular biomarkers that signal NSC and vascular aging, including the overexpression of p16^INK4a [30], telomere shortening, and metabolic stress indicators in stem cell populations. The crosstalk between these signaling pathways is illustrated in Fig. 2. In the vascular system, circulating endothelial progenitor cells (EPCs) [36], VCAM-1, and ICAM-1 have been proposed as biomarkers of endothelial aging and dysfunction.

### 2.2 Vascular dysfunction

Aging of the cerebral vascular system is characterized by a gradual decline in the efficiency of brain tissue perfusion. This is associated with:

• Endothelial dysfunction, resulting in decreased nitric oxide (NO) production and increased vasoconstriction.
• Reduced capillary density, leading to decreased brain oxygenation and the development of ischemia.
• Increased permeability of the blood–brain barrier (BBB), allowing toxic metabolites and pro-inflammatory factors to enter the brain, thereby triggering chronic neuroinflammation [37].

Furthermore, the reciprocal interaction between NSCs and endothelial cells plays a critical role in maintaining neurovascular homeostasis. Senescent endothelial cells, through impaired VEGF and BDNF production, can disrupt the NSC niche, while aging NSCs may contribute to endothelial dysfunction via altered secretory profiles and pro-inflammatory cytokine release. The development of a senescence-associated secretory phenotype (SASP) in both neural and vascular compartments promotes chronic inflammation and neurovascular unit deterioration [31]

These alterations play a critical role in the development of vascular encephalopathy, chronic cerebral ischemia, and stroke. In addition, they contribute to the disruption of the neurovascular unit (NVU), a functional complex comprising neurons, endothelial cells, astrocytes, pericytes, and NSCs, whose coordinated interaction is essential for maintaining brain homeostasis and facilitating neurogenesis.

## 3 Inflammation, oxidative stress, and neurodegeneration

Chronic low-grade inflammation is increasingly recognized as a central mechanism contributing to neurodegeneration in the aging brain. With advancing age, microglial cells — the resident immune cells of the central nervous system — become dysregulated and adopt a pro-inflammatory phenotype. Activated microglia release a range of pro-inflammatory cytokines, including interleukin-6 (IL-6), tumor necrosis factor alpha (TNF-α), and interleukin-1 beta (IL-1β). Sustained expression of these mediators promotes a persistent neuroinflammatory environment, which has been shown to impair the function and survival of neural stem cells (NSCs), disrupt neuronal homeostasis, and contribute to endothelial dysfunction within the cerebral vasculature.

In particular, pro-inflammatory cytokines activate NF-κB signaling in NSCs and glial cells, leading to suppression of neurogenesis, impaired synaptic function, and promotion of cellular senescence [[32], [33], [34]]. The accumulation of senescent glial cells and neurons expressing a senescence-associated secretory phenotype (SASP) further perpetuates chronic inflammation by secreting cytokines, chemokines, growth factors, and matrix metalloproteinases [35].

In parallel, oxidative stress emerges as another critical driver of age-related neurodegenerative processes. The age-associated decline in the cellular antioxidant defense system — particularly the reduction in NADPH levels — impairs the ability to neutralize reactive oxygen species (ROS). As a result, ROS accumulate and inflict widespread oxidative damage to nucleic acids, lipids, and proteins. This oxidative burden compromises mitochondrial integrity and function, further exacerbating energy deficits and initiating apoptotic pathways, ultimately culminating in neuronal loss (Joseph et al., 2013).

Excessive ROS production directly affects mitochondrial DNA integrity and inhibits mitophagy, leading to further mitochondrial dysfunction [21,22]. Oxidative damage to the vascular endothelium also contributes to increased BBB permeability and impaired neurovascular coupling [23,24]. The interaction between oxidative stress and inflammation creates a vicious cycle, amplifying damage to both neural and vascular compartments.

Together, chronic inflammation and oxidative stress form a self-perpetuating cycle that significantly contributes to the pathogenesis of neurodegenerative disorders observed in the elderly population. Inflammaging, the age-related increase in systemic and brain-specific inflammatory activity, further reinforces the chronic pro-degenerative environment in the aging brain.

The interplay between aging, oxidative stress, chronic inflammation, NSC and vascular dysfunction, and neurodegeneration can be summarized through key mechanistic disruptions affecting the neurovascular unit. These mechanisms, along with their consequences and corresponding therapeutic targets, are presented in Table 1.

**Table 1:** Mechanisms and therapeutic targets in neural and vascular aging.

Pathological Mechanism	**Effect on CNS**	**Potential Therapeutic Target**	**References**
**↓ BDNF**	Impaired neuronal survival and synaptic plasticity	BDNF mimetics; exercise	[11]; Wang et al., 2022
**↓ VEGF**	Reduced angiogenesis and neurogenesis; cerebral hypoxia	VEGF stimulators; hypoxic training	[9,10]
**↑ mTOR activity**	Inhibited autophagy; accumulation of cellular damage	mTOR inhibitors (rapamycin, metformin)	[13,19,20]
**↓ AMPK activity**	Reduced energy homeostasis; mitochondrial dysfunction	AMPK activators (resveratrol, exercise)	[25,26]
**↑ Oxidative stress (↓ NADPH)**	DNA/protein/lipid damage; mitochondrial injury	Antioxidants (CoQ10, alpha-lipoic acid)	[21,27,28]
**Endothelial dysfunction**	Impaired vasodilation; reduced perfusion	NO donors; endothelial support	Barrios et al., 2017; [7]
**↓ Capillary density**	Reduced oxygen/nutrient delivery to brain tissue	Angiogenic therapy	[8]
**↑ BBB permeability**	Neuroinflammation and entry of toxins into CNS	BBB stabilizers; anti-inflammatory agents	Lacoste et al., 2024; [24]
**Chronic inflammation**	Disruption of NSC niche; neuronal/glial damage	Cytokine inhibitors (TNF-α, IL-1β blockers)	Heneka et al., 2014; Perry et al., 2010; Salminen et al., 2012

## 4 Antioxidant and anti-inflammatory approaches in the management of age-related cerebrovascular pathology

In the context of brain aging, chronic cerebrovascular dysfunction develops gradually and is often clinically silent for long periods before manifesting as cognitive impairment, mood disturbances, or gait disorders. These slowly progressive cerebrovascular disorders, such as chronic cerebral hypoperfusion, white matter hyperintensities, small vessel disease, and vascular cognitive impairment, are increasingly recognized as major contributors to neurodegeneration in the elderly.

At the core of these conditions lies a combination of oxidative stress, low-grade chronic inflammation, endothelial dysfunction, and disruption of the blood–brain barrier (BBB). These processes create a toxic microenvironment that impairs the neurovascular niche, leading to reduced neurogenesis, astrogliosis, and accumulation of microinfarcts in subcortical regions. The progressive deterioration of cerebral microcirculation also limits the supply of oxygen and nutrients to neural stem cells (NSCs), compromising their survival, proliferation, and differentiation.

• Antioxidant therapy, using compounds such as coenzyme Q10, astaxanthin, and alpha-lipoic acid, aims to counteract the overproduction of reactive oxygen species (ROS), which are elevated in both aging and ischemic states. These agents stabilize mitochondrial function, protect endothelial cells from oxidative injury, and preserve BBB integrity. Alpha-lipoic acid additionally regenerates endogenous antioxidants like glutathione, enhancing the cellular redox balance. By mitigating oxidative damage, antioxidants may help slow the progression of white matter lesions and enhance endogenous repair mechanisms in chronic ischemic conditions.
• Anti-inflammatory strategies, including TNF-α inhibitors, IL-1β antagonists, and emerging immunomodulatory agents, target the persistent neuroinflammatory responses that accompany chronic cerebrovascular disease. Activated microglia and astrocytes perpetuate a pro-inflammatory state that not only damages neurons and glia but also disrupts the function of NSCs and their niche. Modulating this inflammation holds potential to limit secondary neurodegeneration, promote angiogenesis, and restore a more permissive environment for neurovascular regeneration.

An important emerging therapeutic concept is the integration of antioxidant, anti-inflammatory, and stem cell-based strategies into combinatorial regimens. For example, antioxidants may enhance the survival and efficacy of transplanted stem cells by reducing oxidative stress in the neurovascular niche, while anti-inflammatory agents may promote favorable immune microenvironments for stem cell engraftment and repair (Chen et al., 2015; Le Belle et al., 2011; Rybak-Wolf et al., 2021). Such combined approaches offer synergistic potential to restore neurovascular function and slow neurodegeneration.

Importantly, the combination of antioxidant and anti-inflammatory therapies may not only attenuate ongoing injury but also enhance the effectiveness of cell-based treatments, such as mesenchymal stem cell (MSC) transplantation. MSCs exert regenerative effects by secreting trophic factors, modulating inflammation, and supporting vascular and neurogenic repair. Clinical trials exploring MSC-based therapy in vascular dementia and post-stroke cognitive impairment are ongoing, reflecting the growing translational potential of regenerative medicine in neurovascular aging. As of 2023, several early-phase clinical trials are evaluating the safety and efficacy of MSCs in patients with vascular dementia. For example, the study titled "Safety and Efficacy of Umbilical Cord Mesenchymal Stem Cells in Patients With Vascular Dementia" [38] is an interventional Phase 1/2 trial assessing cognitive improvement following intravenous MSC administration.

Induced pluripotent stem cells (iPSCs) offer an additional avenue for regenerative medicine. iPSC-derived neurons and endothelial cells offer a promising in vitro model to study individual aging trajectories and test regenerative compounds in a patient-specific context [39,40]. Their potential in reconstructing neural populations and modeling complex age-related cerebrovascular pathologies positions iPSCs as a cornerstone of future personalized therapies.

Despite encouraging preclinical results, the translation of NSC- and MSC-based therapies into clinical practice remains limited by variability in cell sourcing, delivery methods, and immune compatibility [41,42]. Further research is needed to standardize protocols and ensure reproducibility in human studies.

## 5 Conclusion

The aging of neural stem cells and cerebrovascular systems are interdependent processes that significantly contribute to the pathogenesis of age-related cognitive decline and neurodegeneration. The breakdown of the neurovascular niche, driven by oxidative stress, inflammation, and impaired cellular signaling, leads to a hostile environment that impairs regeneration and accelerates functional deterioration.

Targeting these pathophysiological mechanisms through antioxidant, anti-inflammatory, and stem cell-based therapies offers a multifaceted strategy for neuroprotection and regeneration. Future research should focus on integrating these approaches into combinatory therapeutic regimens, validating their efficacy in clinical settings, and tailoring interventions to individual patient profiles through personalized medicine. This holistic approach holds promise for mitigating the burden of age-related cerebrovascular and neurodegenerative diseases.

## Funding

This research received no external funding.

## CRediT authorship contribution statement

**Khrystyna Duve:** Writing – original draft, Visualization. **Volodymyr Lushchak:** Writing – review & editing, Supervision, Conceptualization. **Svitlana Shkrobot:** Writing – review & editing, Writing – original draft, Methodology.

## Declaration of competing interest

The authors declare no conflict of interest.
